# Influence of Tripolyphosphate on Electronic Conductivity and Photothermal Relaxation Dynamics in Ti_3_C_2_T_*x*_ MXene

**DOI:** 10.1021/acs.jpcc.5c08422

**Published:** 2026-02-06

**Authors:** Andrew M. Fitzgerald, Nikoloz Gegechkori, Laura Londoño Fandiño, Dawei Liu, Kateryna Kushnir Friedman, Joshua R. Uzarski, Ivan Baginskiy, Serhii Dukhnovsky, Veronika Zahorodna, Oleksiy Gogotsi, Ronald L. Grimm, Jeannine M. Coburn, Lyubov V. Titova

**Affiliations:** † Department of Physics, 8718 Worcester Polytechnic Institute, Worcester, Massachusetts 01609, United States; ‡ US Army DEVCOM Soldier Center, Natick, Massachusetts 01760, United States; § 712343 Y-Carbon LLC (Carbon-Ukraine), Kyiv 04116, Ukraine; ∥ 712341 MXene Nano Tech LLC, Philadelphia, Pennsylvania 18974, United States; ⊥ Department of Chemistry and Biochemistry, Worcester Polytechnic Institute, Worcester, Massachusetts 01609, United States; # Department of Biomedical Engineering, Worcester Polytechnic Institute, Worcester, Massachusetts 01609, United States

## Abstract

Ti_3_C_2_T_*x*_ MXene, the most extensively studied member of the MXene family, combines metallic conductivity, strong light absorption, and exceptional photothermal efficiency, enabling applications ranging from optoelectronics to thermal management and biomedical systems. However, its practical use has been challenged by limited environmental stability. While polyphosphate edge-capping has previously been shown to effectively suppress oxidation and degradation in aqueous suspensions, its influence on the intrinsic electronic properties and photothermal behavior of MXene films has remained unexplored. Here, we investigate the impact of sodium tripolyphosphate (TPP) introduced during aqueous processing on the electrical transport and photothermal dynamics of Ti_3_C_2_T_*x*_ films. Using terahertz time-domain spectroscopy (THz-TDS) and four-point probe measurements, we find that TPP addition does not significantly alter charge transport or carrier localization, indicating that the electronic structure of the films is preserved. Optical pump–THz probe spectroscopy reveals that, upon photoexcitation, all samples exhibit the characteristic transient suppression of conductivity associated with photothermal heating, followed by a slow recovery over hundreds of picoseconds. At higher TPP concentrations, the thermal relaxation is noticeably slower, suggesting that TPP residues at flake edges and interflake interfaces hinder phonon transport and heat dissipation. These findings demonstrate that addition of polyphosphate, while maintaining the excellent conductivity of Ti_3_C_2_T_*x*_, can be used to control photothermal relaxation behavior and the thermal response of MXene-based functional materials.

## Graphical abstract

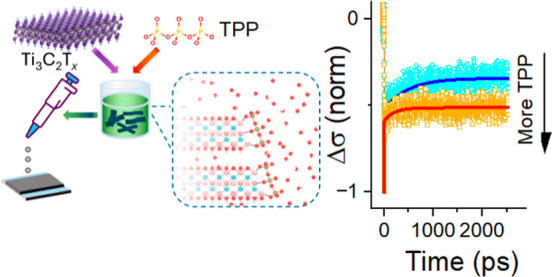

## 1 Introduction

MXenes are a class of layered, two-dimensional (2D) transition metal carbides, nitrides, and carbonitrides that have been gaining significant attention since their discovery in 2011.^1–3^ They share the general chemical formal of M_*n*+1_X_*n*_T_*x*_, where *M* is an early transition metal (such as Ti, Mo, or Nb), *X* is carbon or nitrogen, and *n* takes on a value from 1–4 corresponding to the number of layers in the material. *T*_*x*_ represents the surface terminations that bind to the outermost transition metal layers after the A-layer (usually Al) is selectively etched away from their parent MAX-phase structure via a hydrofluoric chemical treatment in solution.^2–4^ The presence of surface terminations (*T*_*x*_), such as –OH or =O, makes MXenes hydrophilic and facilitates their dispersion in aqueous solutions, enabling easy deposition onto a wide range of substrates. Ti_3_C_2_T_*x*_, a titanium carbide, was the first discovered member of the MXene family and remains the most extensively studied due to high conductivity, conductivity^5–8^ and photothermal conversion efficiency,^9^ record volumetric capacitance,^10,11^ and pronounced optical nonlinearities,^12^ rendering them attractive for a wide-variety of applications including electromagnetic interference (EMI) shielding,^13,14^ flexible electronics,^15^ water purification,^16^ energy storage,^17–19^ optoelectronic and photonic devices,^20,21^ gas sensing,^22,23^ photothermal cancer therapies,^24–28^ and others.

MXenes, particularly Ti-based ones like Ti_3_C_2_T_*x*_, are intrinsically prone to oxidation when exposed to oxygen and moisture, both in aqueous colloidal suspensions and in thin-films stored under ambient conditions with humidity. In aqueous suspensions, Ti_3_C_2_T_*x*_ MXene degrades over time even in oxygen-free environments under an argon atmosphere.^29^ The presence of dissolved oxygen further accelerates oxidation, which typically begins at the edges of MXene flakes and at structural defects,^30,31^ gradually converting Ti_3_C_2_T_*x*_ into insulating titanium oxides. Films exhibit greater stability than colloidal solutions, provided that trapped water is minimized, as the internal flakes are less exposed to oxygen and water, thereby reducing the rates of oxidation and hydrolysis. Since colloidal suspensions are often the starting point for film fabrication and device integration, ensuring their stability is critical to preserving the intrinsic properties of MXenes throughout processing.

Recent efforts have focused on enhancing the oxidative stability of MXenes in aqueous solutions. Strategies include storing colloidal suspensions at subzero temperatures (e.g., −80 °C to −18 °C),^32,33^ and minimizing defect concentrations that can serve as initiation sites for oxidation.^7,8,34^ Another promising approach involves using inorganic polyanionic salts to cap the positively charged edges of individual flakes, thereby preventing interaction with water and oxygen—primary agents of oxidation and hydrolysis. Among these, polyphosphates have shown particular effectiveness: even at concentrations as low as 0.1 M, they can significantly suppress oxidation for over 3 weeks in aerated water at room temperature.^35^ This method not only improves the stability of MXenes in aqueous environments but also offers a scalable, cost-effective, and environmentally friendly solution.^36,37^

While edge-capping techniques have been shown to significantly improve the chemical stability of Ti_3_C_2_T_*x*_, the effects of polyphosphates on the electronic, optical, and photothermal properties of MXene films deposited from solutions containing polyphosphates remain unclear. Here, we present a systematic study of the influence of different concentrations of sodium tripolyphosphate (TPP) in aqueous solution on the electronic and photothermal behavior of Ti_3_C_2_T_*x*_ MXene films. The properties were characterized using terahertz (THz) spectroscopy and conventional four-point probe (4PP) electrical measurements. We find that the addition of TPP does not adversely affect electrical conductivity across a broad range of TPP/MXene mass ratios. However, at a ratio of approximately 100 μg TPP per mg Ti_3_C_2_T_*x*_ or higher, the presence of TPP slows photothermal relaxation following excitation with 800 nm laser pulses. We hypothesize that both edge capping and unbound TPP molecules located between MXene flakes impede phonon transport. Thus, beyond enhancing colloidal stability without compromising conductivity, TPP can be used to fine-tune the thermal relaxation dynamics of MXene films.

## 2 Methods

### 2.1 MAX-Phase and MXene Synthesis

Porous Al–Ti_3_AlC_2_ MAX-phase was synthesized following published procedures.^38^ High-purity Ti_3_AlC_2_ powder (<40 μM, 99% porous; Figure S1a) was washed in 20% HCl at 50 °C for 6 h to remove intermetallic impurities. Ti_3_C_2_T_*x*_ MXene was then prepared by selective wet-chemical etching of 20 g of the washed Ti_3_AlC_2_ in a mixed acid solution (2 wt parts HF (50%), 12 wt parts HCl (36%), and 6 wt parts deionized water; total 400 g) at 35 °C for 24 h. The etched product was repeatedly centrifuged (1900 rcf, 10 min) and redispersed until the supernatant reached pH 5.

Delamination was achieved by Li^+^ intercalation: the rinsed MXene slurry was stirred in 50 g/L LiCl solution (400 mL) at 35 °C for 24 h, followed by centrifugation (1400 rcf, 10 min) and redispersion. The supernatant containing delaminated MXene flakes was stored frozen. Concentrated MXene slurry was obtained by centrifugation (2500 rcf, 20 min); its solid content (∼16 wt %) was determined by drying at 50 °C to constant mass. Concentrated MXene slurry was stored at −20 °C until used.

### 2.2 Polyphosphate/MXene Film Preparation

A 6.4 mg/mL MXene stock solution was made by diluting the MXene slurry in ultrapure water (PicoPure water purification system, Hydro Service and Supplies, Durham, NC) followed by vortexing to create a colloidal suspension. A sodium tripolyphosphate solution (TPP, Sigma-Aldrich, St. Louis, MO) was prepared at 5 mg/mL in ultrapure water. The TPP solution was serial diluted 1:1 in ultrapure water to a concentration of 0.039 mg/mL (7 serial dilutions). MXene working solution and the individual TPP solutions were mixed 1:1 to obtain 780, 390, 200, 98, 49, 24, 12, or 6 μg TPP: 1 mg MXene solutions. For the pure MXene controls, the MXene working solution was diluted 1:1 in water to obtain a solution with the same final MXene concentration. The solutions were further diluted using ultrapure water to a final MXene concentration of 0.8 mg/mL. For UV–vis, transient absorption (TA) spectroscopy, THz spectroscopy, and electronic four-point probe measurements, 50 μL MXene containing solutions were drop-casted onto 1 mm thick quartz (FireflySci, Inc., Northport, NY) and allowed to air-dry (2–4 h). The area of sample coverage was standardized to approximately 0.2 cm^2^. Film thickness was measured using a profilometer and is listed in Table S1. For scanning electron microscopy (SEM), 10 μL MXene containing solutions were drop-casted onto prescored N-type (P-doped) silicon substrates (UniversityWafer, Inc., Boston, MA). After drying, the silicon substrates were cracked down the score line to allow for cross-sectional imaging.

#### 2.2.1 Scanning Electron Microscopy

The morphology of the samples was characterized by SEM (JEOL-7000F, JEOL Inc., Peabody, MA). Samples were mounted on a 45°-tilted holder, and images were captured under high vacuum with an accelerating voltage of 5 kV.

### 2.3 UV–vis Spectroscopy

A UV–Vis spectrometer (Evolution 300, Thermo Fisher Scientific, Waltham, MA) was used to collect UV–Vis spectra of the MXene films in transmission mode.

### 2.4 Terahertz Spectroscopy

The intrinsic complex conductivity of Ti_3_C_2_T_*x*_ films was characterized over the 0.25–2.5 THz frequency range using terahertz time-domain spectroscopy (THz-TDS) in transmission mode. Measurements were performed with a TeraFlash Pro spectrometer (Toptica Photonics). THz-TDS is a noncontact, all-optical technique that enables frequency-resolved analysis of complex conductivity by capturing both the amplitude and phase of THz pulses transmitted through the sample (MXene film on substrate) and a reference (bare substrate).^39,40^ Complex conductivity is calculated using the Tinkham thin-film approximation

1 $$\frac{{Ẽ}_{sample}\left(\omega \right)}{{Ẽ}_{substrate}\left(\omega \right)}=\frac{n+1}{n+1+Z_{0}\mathrm{\sigma ̃}\left(\omega \right)d}$$

where *n* is the refractive index of the fused quartz substrate in the THz frequency range (1.95 ± 0.05 within the 0.25–2.5 THz range),^41^ *Z*_0_ is the impedance of free space (377 Ω), *d* is the film thickness, and ${Ẽ}_{sample}\left(\omega \right)$ and ${Ẽ}_{substrate}\left(\omega \right)$ are the electric fields of the THz pulses transmitted through the sample-on-substrate and the bare substrate, respectively alone.

To investigate photoinduced changes in conductivity, Ti_3_C_2_T_*x*_ films were studied using optical-pump THz-probe (OPTP) spectroscopy with 1.55 eV (800 nm), 100 fs laser pulses for excitation. A custom-built setup was employed, generating THz probe pulses via a 1 mm-thick [100]-oriented ZnTe crystal excited by 800 nm, 100 fs pulses from an amplified Ti/sapphire laser. The resulting THz pulses (1–10 meV bandwidth) were focused onto the sample using off-axis parabolic mirrors, forming a ∼1.5 mm spot at normal incidence. Transmitted THz pulses were collected and detected via electro-optic sampling in a second ZnTe crystal. Optical excitation was delivered through a 5 mm aperture in the focusing mirror, producing a ∼5 mm spot to ensure uniform illumination across the THz probe area. A mechanical delay line controlled the relative timing between pump and probe pulses for time-resolved conductivity measurements.

### 2.5 Four-Point Probe Electrical Measurements

The DC conductivity of the samples was measured using a custom four-point probe (4PP) setup. A Keysight B2911A Precision Source/Measure Unit (Santa Rosa, CA) was used to supply current and measure voltage. The setup utilized four DCP 100 Series probes with tungsten tips (DPP220) spaced apart by 1 mm. Current was driven through the outer probes, while the inner probes detected the resulting voltage drop, allowing for current–voltage (*I*–*V*) data collection. The sheet resistance was calculated using the thin-film approximation $\rho_{sq}=\frac{\pi }{\ln\left(2\right)}\frac{\Delta V}{I}$ , and DC conductivity was calculated as $\sigma_{DC}={\left(\rho_{sq}\cdot d\right)}^{-1}$ , where *d* is the sample thickness.^42^

## 3 Results and Discussion

To evaluate the influence of TPP on the functional properties of Ti_3_C_2_T_*x*_ films, we examined samples prepared from aqueous solutions with varying TPP/MXene mass ratios, ranging from pure MXene to 780 μg TPP per mg Ti_3_C_2_T_*x*_, as described in the Methods section and illustrated schematically in Figure 1a. Representative SEM images are shown in Figure 1b,c, with Figure 1b depicting a pure Ti_3_C_2_T_*x*_ film and Figure 1c showing a film prepared from a solution containing 200 μg TPP per mg Ti_3_C_2_T_*x*_. Additional lower-resolution SEM images are provided in Figure S1. The films exhibit considerable thickness nonuniformity and loosely packed flakes, which are characteristic of the drop-casting process. This is further supported by stylus profilometry measurements (Table S1), which show surface roughness values in the range of 80–140 nm. SEM images reveal that films prepared with TPP exhibit additional local surface texture compared to pure MXene films. However, because this local roughness occurs on smaller length scales than the overall film nonuniformity inherent to drop-casting, it is not captured in the profilometry data. We hypothesize that this fine-scale surface roughness arises from TPP disrupting or altering interflake interactions during film formation.

**Figure 1:**
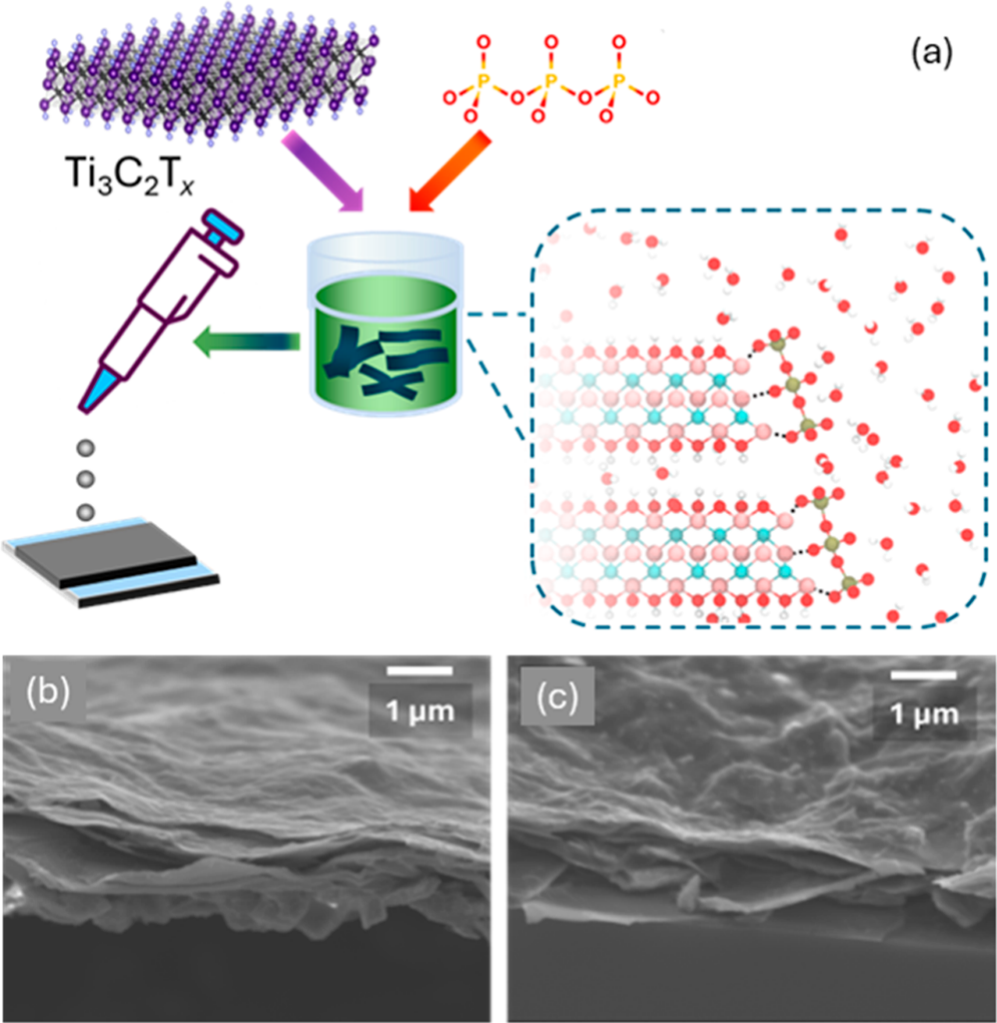
(a) Ti_3_C_2_T_*x*_ is combined with triphosphate to cap flake edges and improve stability before drop-casting on a quartz substrate. SEM images of (b) a pure sample of Ti_3_C_2_T_*x*_ and (c) a sample prepared using a solution of 200 μg TPP/mg Ti_3_C_2_T_*x*_.

Figure 2 presents the UV–vis spectra of all studied films, normalized to 264 nm peak and offset for clarity. Each spectrum exhibits a broad absorbance peak centered around 780 nm, a feature characteristic of Ti_3_C_2_T_*x*_. This peak has been attributed in the literature to either optically active localized surface plasmon resonance (LSPR) or interband transitions.^58–202^ Notably, the position and width of this peak remain unchanged across all TPP concentrations, indicating that the electronic and optical properties of Ti_3_C_2_T_*x*_ are preserved upon TPP addition.

**Figure 2:**
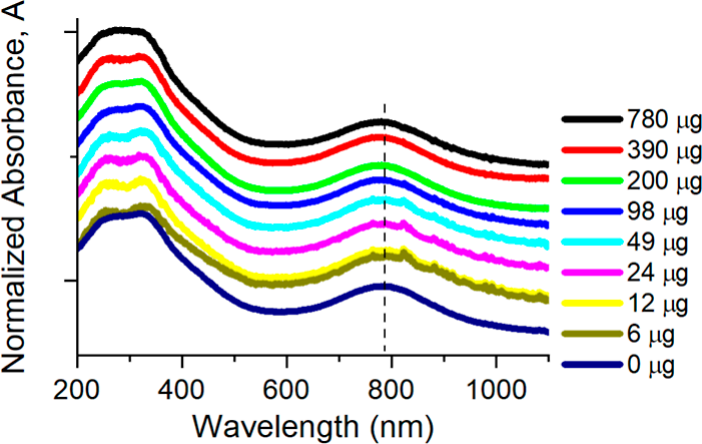
UV–vis spectra of pure Ti_3_C_2_T_*x*_ and Ti_3_C_2_T_*x*_ with TPP films in concentrations per 1 mg of Ti_3_C_2_T_*x*_ indicated in the legend, normalized to the peak at 264 nm. Spectra are vertically offset for clarity. A broad absorbance peak attributed to an interband transition is observed at ∼788 nm for each sample, as indicated by a dashed vertical line.

Figure 3 summarizes the electronic properties of Ti_3_C_2_T_*x*_ films, presenting both the complex conductivity extracted from THz time-domain spectroscopy (THz-TDS) and the current–voltage (*I*–*V*) characteristics measured via four-point probe (4PP) for two representative samples: a pure Ti_3_C_2_T_*x*_ film (Figure 3a,b) and a film prepared with a 200 μg TPP: 1 mg MXene mass ratio (Figure 3c,d). Complete data sets of THz-TDS spectra and 4PP *I*–*V* curves are provided in Figures S2 and S3. The THz-TDS spectra reveal a suppression of the real part of conductivity at lower frequencies and negative imaginary conductivity, consistent with carrier backscattering and restricted motion over nanometer-scale distances, observed in nanogranular metals.^43,44^

**Figure 3:**
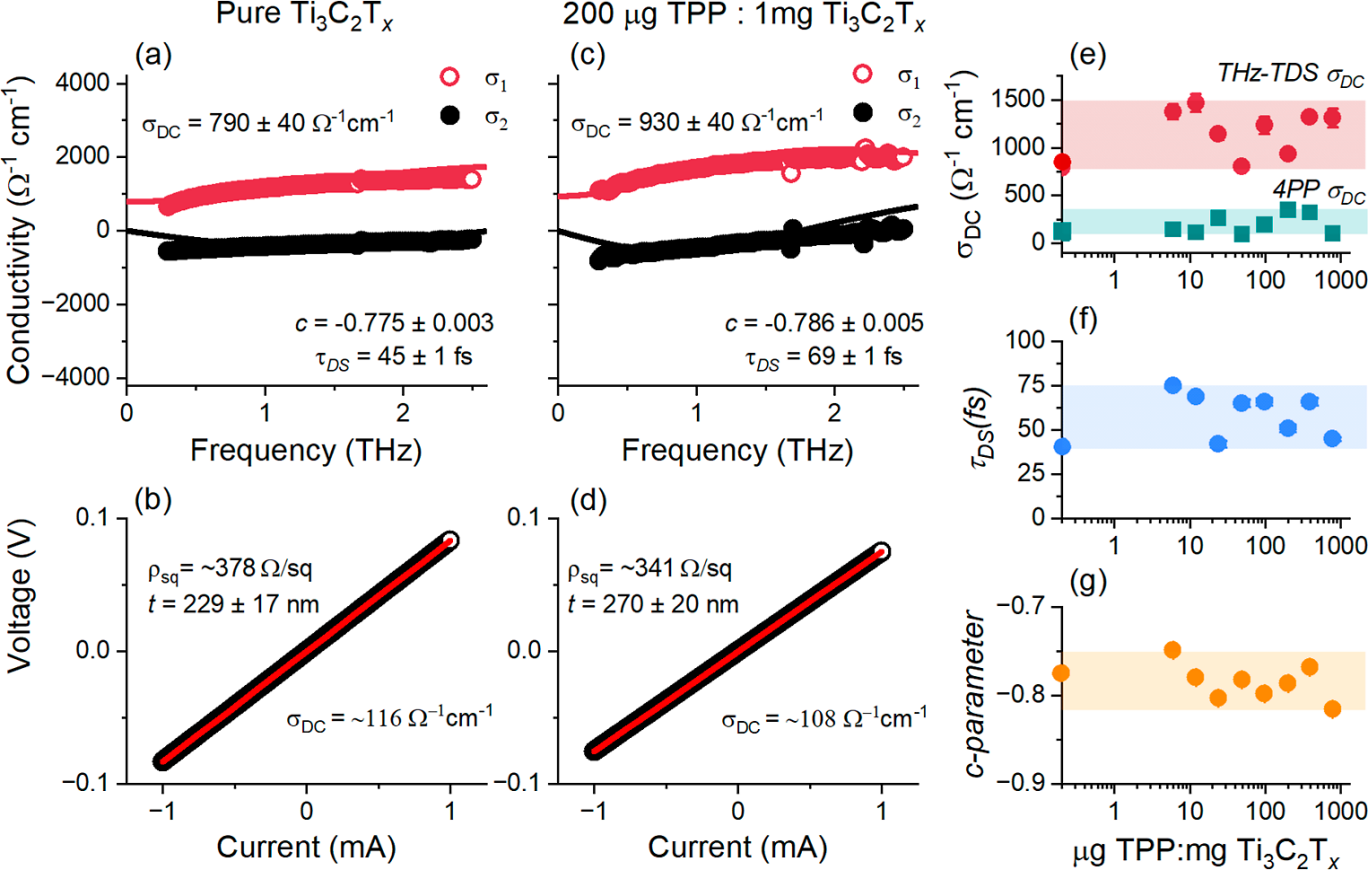
THz-TDS spectra and *I*–*V* characteristics of Ti_3_C_2_T_*x*_ films: (a,b) pure Ti_3_C_2_T_*x*_; (c,d) film with 200 μg TPP per mg Ti_3_C_2_T_*x*_. In (a,c), symbols show real (σ_1_) and imaginary (σ_2_) conductivity; solid lines are Drude–Smith fits. In (b,d), symbols are experimental *I*–*V* data; red lines are linear fits for sheet resistance and conductivity. (e) DC conductivity from 4PP (teal) and THz-TDS (red); (f) scattering time and (g) c-parameter vs TPP/Ti_3_C_2_T_*x*_ ratio. Shaded regions indicate parameter variation.

This behavior is characteristic of MXenes and has been previously observed in Ti_3_C_2_T_*x*_, Mo_2_Ti_2_C_3_T_*x*_, Mo_2_TiC_2_T_*x*_, Nb_4_C_3_T_*x*_, and Nb_2_CT_*x*_, where it is ascribed to disorder and effects of flake boundaries.^45–51^ The observed frequency-dependent conductivity is well described by the Drude–Smith model,^52,53^ a modification of the classical Drude model that accounts for carrier localization effects and reflects an ensemble-averaged response over the film containing many loosely packed flakes (Figure 1b,c). In this framework, the complex conductivity as a function of angular frequency ω, is given by

2 $$\mathrm{\sigma ̃}\left(\omega \right)=\frac{\sigma_{0}}{1-i\omega \tau_{DS}}\left(1+\frac{c}{1-i\omega \tau_{DS}}\right)$$

where $\sigma_{0}=\frac{Ne^{2}\tau_{DS}}{m^{*}}$ is the Drude weight, τ_DS_ is carrier scattering time, *N* is the charge carrier density, m* is the carrier effective mass, and c is localization parameter ranging from 0 (free carrier motion) and −1 (complete localization). The DC conductivity, σ_DC_ = σ_0_(1 + *c*) can be estimated by extrapolating the real part of the Drude-Smith model to *f* = 0 THz. The Drude-Smith parameters (σ_DC_, τ_DS_, and *c*) for all films are given in Figure 3e–g. Figure 3e also shows DC conductivity determined using 4PP measurements.

First, although some variation in the measured parameters is observed, all values remain within relatively narrow ranges, as indicated by the shaded regions in Figure 3e–g. This demonstrates that even at the highest TPP concentration (780 μg: mg MXene), the electronic properties of the films are not appreciably altered, with the observed variations attributed to the inherent inhomogeneity of drop-cast films. Across all samples, from pure MXene to those with the highest TPP content, the DC conductivity extracted from THz spectra remains within σ_DC,THz_ = 1150 ± 340 Ω^–1^ cm^–1^. The *c*-parameters lie in the range of −0.75 to −0.81, indicating significant carrier localization, while the carrier scattering time ranges from 45 to 75 fs.

From these scattering times, the intrinsic (short-range) carrier mobility within individual nanoflakes can be estimated as $\mu_{short-range}=\frac{e\tau_{DS}}{m^{*}}$ . Using m* = 0.2845 m_e_,^54,55^ we estimate the short-range carrier mobility to be in the range of 280–460 cm^2^ V^–1^ s^–1^. The long-range carrier mobility, which accounts for the effects of localization over length scales of tens of nanometers and is scaled by the factor (1 + c), is significantly lower, falling within the range of ∼60–90 cm^2^ V^–1^ s^–1^.

The DC conductivity of the films measured via 4PP, σ_DC,4pp_, is approximately 5–10 times lower than the conductivity extracted from THz-TDS measurements. This discrepancy arises from the different length scales probed by the two techniques. THz-TDS captures the short-range, microscopic conductivity dominated by intraflake carrier transport over lengths scales <50 nm, averaged across many flakes within the ∼1.5 mm diameter of the THz probe beam.^56^ In contrast, the 4PP method measures macroscopic conductivity over a 3 mm distance (based on 1 mm probe spacing), which is more sensitive to film inhomogeneities, such as flake boundaries, voids, and thickness variations inherent to drop-cast films. This observation is consistent with prior electrical transport studies, where the conductivity of multilayer Ti_3_C_2_T_*x*_ films was found to be approximately 1 order of magnitude lower than that of individual flakes, indicating relatively efficient interflake charge transport despite the presence of surface terminations·^57^ Similar to the THz-derived DC conductivity, the 4PP-measured DC conductivity shows no clear trend with TPP concentration and remains within σ_4PP_ = 190 ± 100 Ω^–1^ cm^–1^. This consistency confirms that the electronic properties of the Ti_3_C_2_T_*x*_ films are largely unaffected by the presence of TPP.

Finally, Figure 4 illustrates how the THz conductivity of the films is affected by optical excitation using 100 fs, 800 nm laser pulses with a fluence of 950 μJ cm^–2^. Figure 4a,b show the normalized change in the peak transmission of the THz probe pulse—representative of the overall transmission across the 0.5–2.5 THz bandwidth—as a function of delay time after photoexcitation for two selected samples with TPP: Ti_3_C_2_T_*x*_ ratios of 24 μg: 1 mg and 200 μg: 1 mg, respectively, presented over two different time windows. The corresponding data for all films are provided in Figure S4.

**Figure 4:**
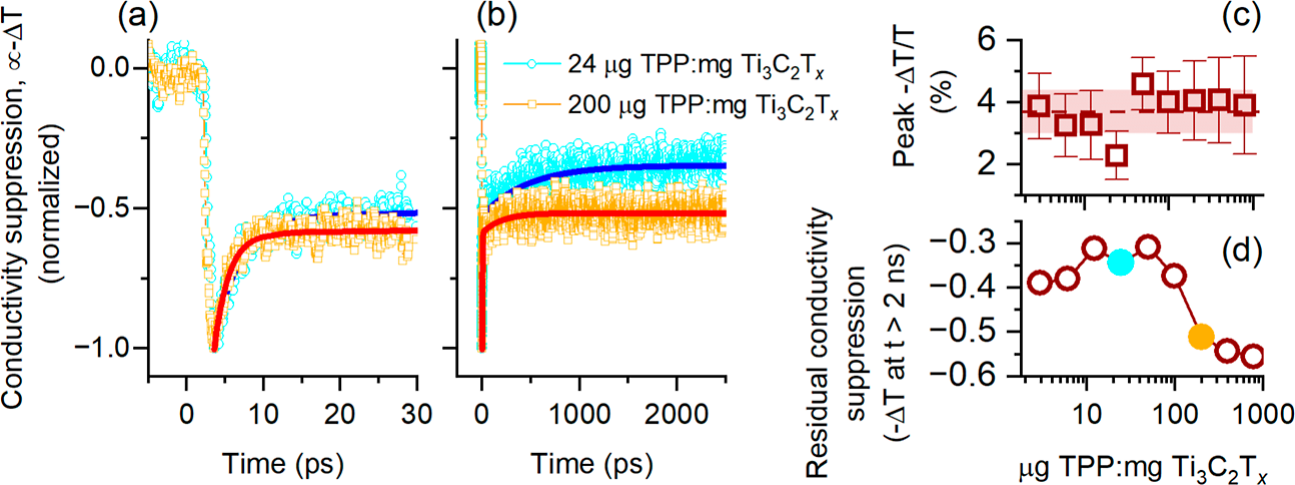
(a,b) Photothermal conductivity suppression in Ti_3_C_2_T_*x*_ films upon 800 nm photoexcitation: (a, b) normalized THz transmission change (proportional to conductivity) for samples with TPP/Ti_3_C_2_T_*x*_ ratios of 24 μg: 1 mg and 200 μg/mg shown over different time windows. Symbols: data; solid lines: biexponential fits. (c) Peak of conductivity suppression vs TPP/Ti_3_C_2_T_*x*_ ratio. Shaded region represents parameter variation. (d) Offset parameter from biexponential fits, representing residual thermal conductivity suppression at long times (>2 ns), relative to the peak suppression shown in (a,b), vs TPP/Ti_3_C_2_T_*x*_ ratio.

Changes in THz transmission through thin conductive films following optical excitation arise when the excitation alters film conductivity. For small changes (<20%), a negative change in the THz peak transmission is proportional to photoconductivity, −Δ*T*(*t*)∝Δσ­(*t*).

Consistent with previous reports on photoexcited Ti_3_C_2_T_*x*_,^46–48,50,51,58^ absorption of the laser pulse results in a rapid increase in THz transmission, indicating a transient suppression of conductivity. In a previous study on Ti_3_C_2_T_*x*_, we demonstrated that optical excitation leads to a suppression of the THz conductivity across the entire experimental frequency range (0.5–2.5 THz).^50^ In MXene films, optical excitation interacts with carriers through two primary pathways: (i) absorption by intrinsic free carriers, generating a hot carrier population, and (ii) interband excitation of additional carriers. For highly conductive Ti_3_C_2_T_*x*_ with intrinsic carrier densities exceeding 10^20^ cm^–3^, the intraband process dominates, leading to conductivity suppression. The photoexcited hot carriers transfer their excess energy to the lattice within <300 fs, resulting in efficient photothermal conversion and a corresponding rise in lattice temperature that reduces metallic conductivity.^50,51,58,59^

Here, we find that the magnitude of the initial change in THz peak transmission (−Δ*T*/*T*)—which reflects the lattice temperature increase—is approximately 4% at the excitation fluence used and remains essentially unchanged across all TPP concentrations (Figure 4c), indicating that the addition of TPP does not affect the initial photoinduced processes, such as the rapid lattice heating within the MXene flakes. Following this initial suppression, the recovery kinetics of the THz transmission capture thermal relaxation processes. Comparison of these transient recovery dynamics thus provides insight into the influence of TPP on heat dissipation and carrier–phonon coupling.

As in previous studies of drop-cast Ti_3_C_2_T_*x*_ films,^56^ the recovery dynamics within the studied time window (<2.5 ns) are well described by a biexponential decay with a constant offset, representing slower processes extending beyond this time frame. The fast and intermediate decay times for films with varying TPP loadings are presented in Figure S5, which shows that—apart from variations attributable to film inhomogeneity—these times remain unchanged across the entire range of TPP concentrations. The fast component (τ_1_ = 3.7 ± 1.5 ps) corresponds to hot carrier cooling, while the slower intermediate component (τ_2_ = 500 ± 190 ps) describes heat transfer and thermal relaxation across the film. A parameter that does show a pronounced change is the slowest relaxation component (>2 ns), which cannot be reliably extracted as a time constant due to the limited 2.5 ns experimental time window. Instead, it appears in the biexponential fit as a constant offset, representing the residual transient conductivity suppression observed at long times (>2 ns) after excitation, relative to the normalized peak suppression (Figure 4a,b). As shown in Figure 4d, the long-time residual conductivity suppression—indicative of elevated lattice temperature—remains at ∼ 30–40% of its peak value at for lower TPP concentrations, consistent with previous results for pure Ti_3_C_2_T_*x*_ films. However, at higher TPP loadings (>100 μg:1 mg MXene), the long-time component increases, with the lattice temperature remaining at 50–55% of its peak value at 2.5 ns, indicating further slowing of thermal relaxation. This behavior contrasts with earlier findings in silk–MXene composites,^56^ where the addition of silk accelerated thermal relaxation due to efficient heat transfer from MXene flakes to silk fibroin. As shown in the SEM images (Figure 1b,c), films containing TPP exhibit additional fine-scale surface texture, suggesting that TPP modifies local film morphology by altering flake packing and interflake connectivity. While overall surface roughness remains high due to the drop-casting process, the presence of TPP introduces localized structural disorder—likely through its attachment to flake edges and defects—which disrupts interflake interactions. This disruption hinders phonon coupling between adjacent MXene flakes and introduces additional phonon scattering sites at interfaces. Collectively, these microstructural changes reduce the continuity of thermal pathways through the film, manifesting as slower thermal relaxation following photoexcitation.

## 4 Conclusions

In this study, we investigated how the addition of polyphosphate salts, specifically sodium tripolyphosphate (TPP), used to stabilize Ti_3_C_2_T_*x*_ MXene flakes against oxidation and hydrolysis in aqueous solutions, affects the electronic and photothermal properties of films deposited from these solutions. By characterizing film conductivity using both traditional four-point probe (4PP) measurements and THz-TDS, we found that the electronic properties of the films remain largely unaffected by the presence of TPP. This highlights TPP as an effective and low-cost strategy for enhancing MXene stability without compromising electrical performance.

We also examined the impact of TPP on the thermal relaxation dynamics of Ti_3_C_2_T_*x*_ following optical excitation, using optical pump-THz probe spectroscopy. Using the photothermal suppression of THz conductivity as an indicator of lattice temperature allowed us to monitor thermal relaxation. At sufficiently high TPP concentrations, we observed a slowdown in thermal relaxation, likely due to hindered phonon transport between flakes as TPP residues alter flake packing density and interflake connectivity. This effect builds upon the already known slow thermal relaxation in Ti_3_C_2_T_*x*_ and suggests a potential pathway for tuning photothermal behavior. Such control over thermal relaxation could be valuable for applications in photothermal therapy and thermal energy storage.

## Supplementary Material



## Notes

SEM images; film thicknesses measured by profilometer; complex THz conductivity spectra, I–V characteristics, and transient photoconductivity decays for all studied TPP/Ti_3_C_2_T_*x*_ ratios; biexponential fit parameters: fast and intermediate relaxation times (PDF)
