# Supplementary material for: Influence of Tripolyphosphate on Electronic Conductivity and Photothermal Relaxation Dynamics in Ti3C2T x MXene
Source: J Phys Chem C Nanomater Interfaces. 2026 Feb 6;130(7):2660–7. doi: 10.1021/acs.jpcc.5c08422 (PMC12927013; doi:10.1021/acs.jpcc.5c08422)
Supplement: Supplementary file 1 [file jp5c08422_si_001.pdf]

Influence of Tripolyphosphate on Electronic Conductivity and Photothermal Relaxation Dynamics in $Ti_3C_2T_x$ MXene

Andrew M. Fitzgerald[1], Nikoloz Gegechkori[1], Laura Londoño Fandiño[1], Dawei Liu[1], Kateryna Kushnir Friedman[1], Joshua R. Uzarski[2], Ivan Baginskiy[3], Serhii Dukhnovsky[3], Veronika Zahorodna[3,4], Oleksiy Gogotsi[3],[4], Ronald L. Grimm[5], Jeannine M. Coburn[6*], Lyubov V. Titova[1*]

[1] Department of Physics, Worcester Polytechnic Institute, Worcester, MA, 01609, USA

[2] US Army DEVCOM Soldier Center, Natick, MA, 01760, USA

[3] Y-Carbon LLC (Carbon-Ukraine), Kyiv, Ukraine

[4] MXene Nano Tech LLC, Philadelphia, PA, USA

[5] Department of Chemistry and Biochemistry, Worcester Polytechnic Institute, Worcester, MA, 01609, USA

[6]Department of Biomedical Engineering, Worcester Polytechnic Institute, Worcester, MA, 01609, USA

*Co-Corresponding Authors: Jeannine M. Coburn (jmcoburn@wpi.edu), Lyubov V. Titova (ltitova@wpi.edu)

Supporting Information

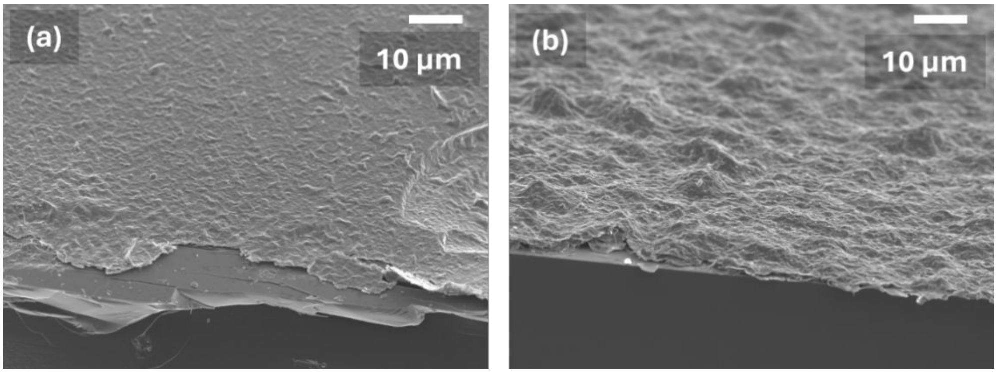

Figure S1: Scanning Electron Microscopy (SEM) images of **(a)** a pure sample of $Ti_3C_2T_x$ and **(b)** a sample prepared using a solution of 200 µg TPP:mg $Ti_3C_2T_x$.

Table S1: Thickness and surface roughness of the thin-films after deposition via drop-casting on quartz substrates as measured via stylus profilometry using the KLA Tencor AlphaStep D-600 Stylus Profilometer.

**Solution used to prepare sample** (**µg TPP:mg $Ti_3C_2T_x$**)	**Thin-film thickness after deposition** (**nm**)	**Thin-film surface roughness after deposition** (**Root Mean Square Roughness, nm**)
0	270 ± 20	130
6	166 ± 13	116
12	157 ± 42	95
24	173 ± 13	80
49	213 ± 24	120
98	199 ± 43	115
200	229 ± 17	140
390	213 ± 45	118
780	224 ± 13	136

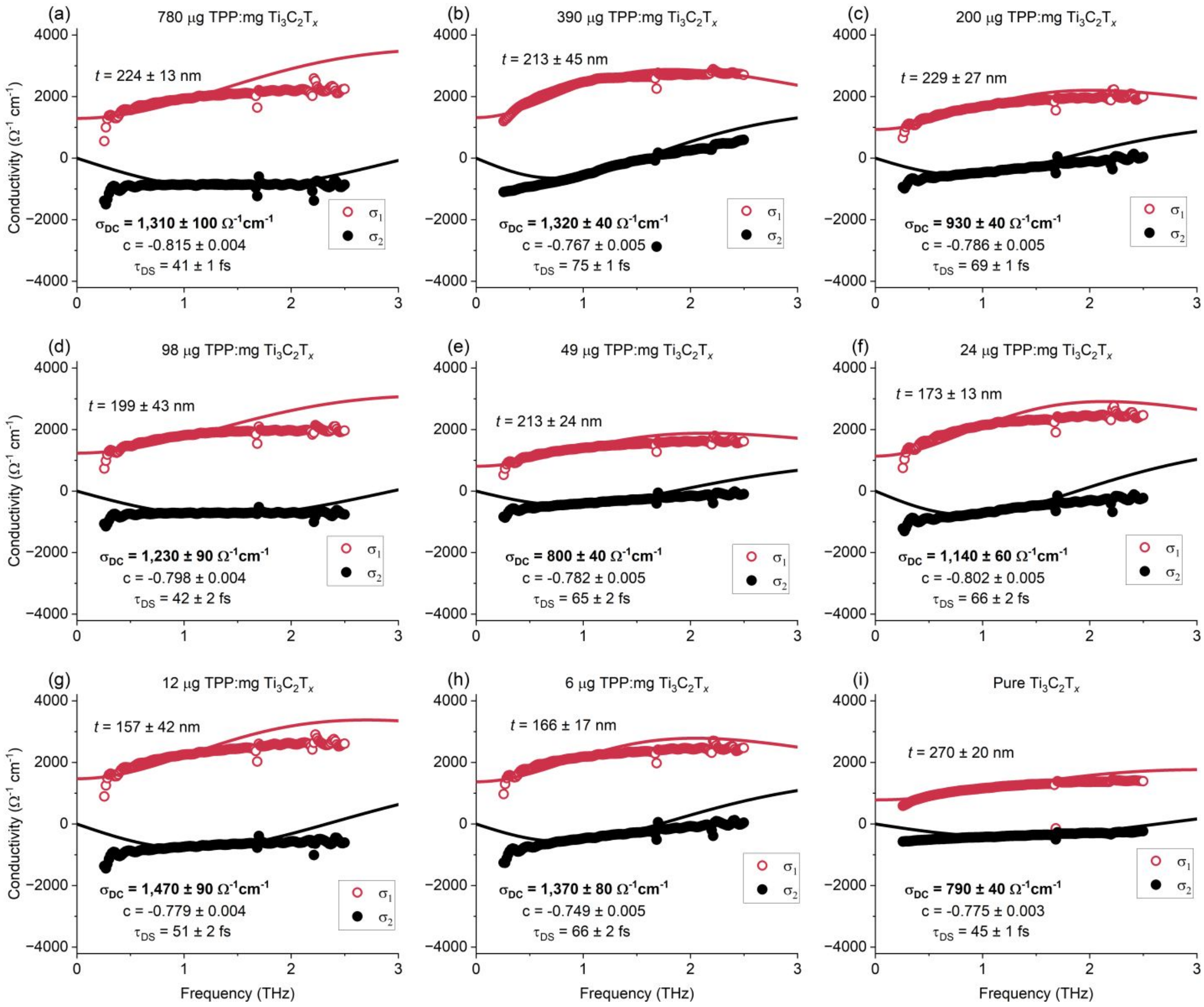

Figure S2: Complex THz conductivity spectra for all studied TPP:$Ti_3C_2T_x$ ratios (as indicated). Symbols: experimental data; lines: Drude–Smith fits with parameters shown in panels.

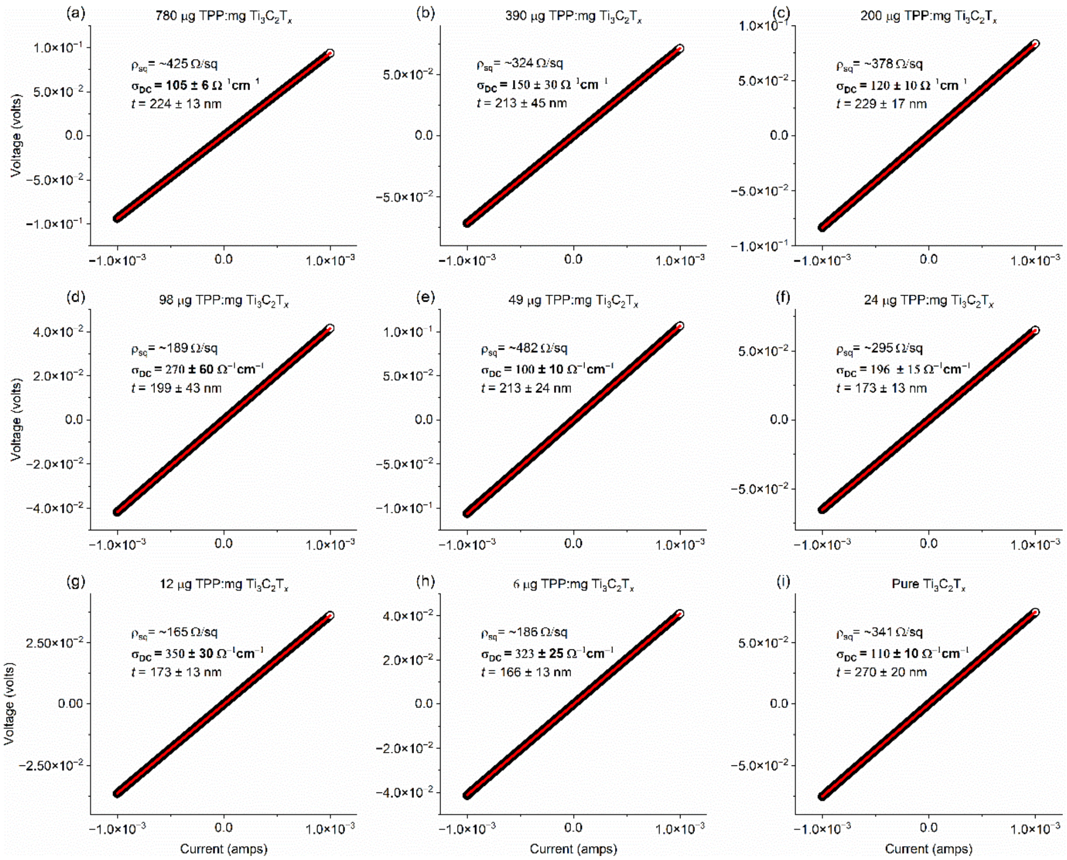

Figure S3: I-V characteristics for all studied TPP:$Ti_3C_2T_x$ ratios (as indicated). Symbols are experimental I–V data; red lines are linear fits for sheet resistance and conductivity, which are given in the panels.

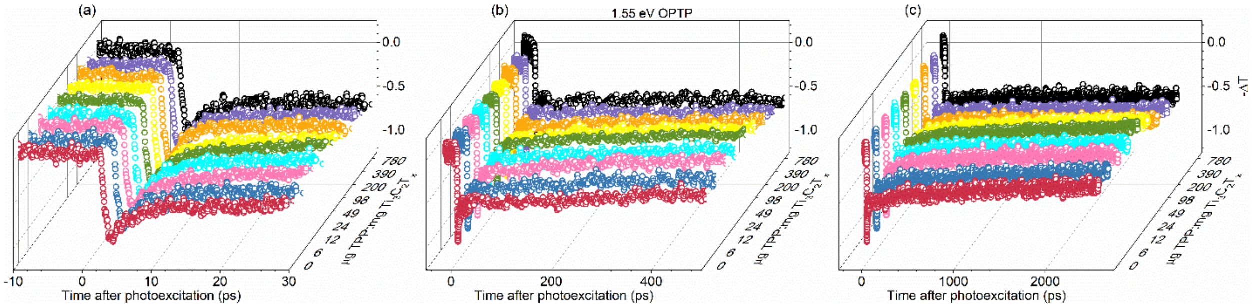

Figure S4: OPTP photoconductivity measurements for all studied TPP:$Ti_3C_2T_x$ ratios (as indicated). Each panel shows the same data in a different time window.

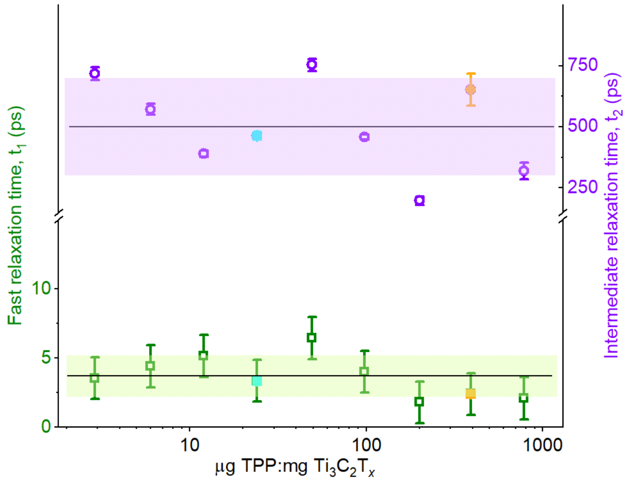

Figure S5: Fast and intermediate relaxation times extracted from bi-exponential fits of OPTP decay curves as a function of the TPP:$Ti_3C_2T_x$ ratio. The solid line indicates the mean value of each parameter, and the shaded region represents the standard deviation from the mean.
